# Ultrasound combined with microbubbles promotes diabetic wound healing by regulating macrophage polarization

**DOI:** 10.3389/fendo.2026.1781906

**Published:** 2026-03-06

**Authors:** Huabin Yang, Qian Feng, Nan Huang, Wen Zhang, Tao Jiang, Rui Wang, Linling Li, Jie Tao, Juan Tang, Zhong Chen

**Affiliations:** 1 Department of Ultrasound, The General Hospital of Western Theater Command, Chengdu, Sichuan, China; 2 Clinical Biobank Center and Laboratory Animal Center, The General Hospital of Western Theater Command, Chengdu, Sichuan, China

**Keywords:** diabetes mellitus, macrophages, transcriptomics, ultrasonic cavitation therapy, wound healing

## Abstract

**Background:**

One of the crucial reasons for the impaired healing of diabetic wounds is the excessive and prolonged inflammatory response at the wound site, which leads to the persistent accumulation of neutrophils and M1 macrophages, the release of abundant pro-inflammatory cytokines, and the disruption of the balance in macrophage polarization. Macrophages, as central regulators of inflammatory responses, play a pivotal role in wound healing impairment through their polarization states. This study aimed to investigate the therapeutic effect of low-intensity ultrasound combined with microbubbles (USMB) on diabetic wound healing and the molecular mechanisms by which USMB regulates macrophage polarization.

**Methods:**

Diabetic rat wound models were established, and rats with successful modeling were divided into three groups: the Model group, the Ultrasound (US) group, and the Ultrasound Combined with Microbubbles (USMB) group. Wound repair efficacy was evaluated by calculating the wound healing rate, conducting histological examinations (HE and Masson staining), and performing immunohistochemical staining(CD31,Ki67).Macrophage polarization was detected using immunofluorescence staining (CD86, CD206) and qRT-PCR (pro-inflammatory/anti-inflammatory factors). Transcriptomic sequencing was conducted on wound tissues from the USMB and Model groups on day 6, followed by validation of key pathway molecules using qRT-PCR and Western blotting.

**Results:**

USMB treatment accelerated wound healing, enhanced granulation tissue formation, increased collagen deposition, stimulated cell proliferation, and promoted angiogenesis. Meanwhile, the USMB group exhibited decreased expression of M1-type macrophage marker CD86 and pro-inflammatory cytokines (IL-1β, IL-6), along with increased expression of M2-type marker CD206 and anti-inflammatory cytokines (IL-10, Arg1). Transcriptomic analysis identified 1725 differentially expressed genes between the two groups, with the IL-17 signaling pathway being significantly enriched. Further validation revealed that the mRNA and protein expressions of pathway-related molecules including IL-17B, NF-κB, and TNF-α in the USMB group were significantly lower than those in the Model group.

**Conclusion:**

USMB treatment can promote wound healing in diabetic rats, and its potential mechanism may lie in the targeted inhibition of the IL-17 signaling pathway, particularly acting on the IL-17B/NF-κB/TNF-α axis. This thereby promotes the polarization of macrophages from the M1 to M2 phenotype, reduces excessive inflammatory responses, improves the inflammatory microenvironment, and further accelerates tissue repair and regeneration.

## 1 Introduction

Diabetes mellitus is a metabolic disorder. According to the International Diabetes Federation (IDF), around 500 million adults worldwide currently have diabetes, a figure projected to rise to 780 million by 2045 (1). Diabetic foot ulcers (DFUs) are among the most severe late-stage complications of diabetes, occurring in 19%–34% of patients. Approximately 30% of these cases result in amputation due to delayed or insufficient treatment (2, 3). Unlike acute wounds, which heal through orderly phases of inflammation, proliferation, and remodeling, DFUs are characterized by a prolonged inflammatory phase that disrupts subsequent angiogenesis and matrix reconstruction (4, 5). In recent years, several novel therapeutic strategies targeting the inflammatory microenvironment of diabetic wounds have been proposed. For instance, additives such as antibacterial hydrogels and immunomodulatory cytokines have been incorporated into existing dressing materials to enhance therapeutic efficacy (6). However, technical limitations hinder the clinical translation and development of such interventions. Furthermore, the therapeutic effects of these novel approaches have failed to be reproducible in clinical settings, limiting their translational potential.

Regulation of macrophage phenotypic homeostasis is the core mechanism underlying wound healing, where the classical activation (M1) and alternative activation (M2) phenotypes exhibit a dynamic balance in spatial and temporal dimensions. Functional studies have demonstrated that M1 macrophages secrete pro-inflammatory cytokines such as IL-6, IL-1β, and TNF-α to exacerbate inflammatory responses, while M2 macrophages release inflammatory mediators including IL-10 and Arg1 to exert anti-inflammatory effects, and secrete vascular endothelial growth factor (VEGF) to promote wound healing (7). In classical physiological wound healing, the monocyte-macrophage system achieves a dynamic balance between the timely resolution of inflammation and the ordered activation of tissue repair through the sequential polarization from M1 (pro-inflammatory phenotype) to M2 (pro-reparative phenotype) (4). However, in the diabetic microenvironment, processes such as hyperglycemia-mediated oxidative stress and abnormal accumulation of advanced glycation end products (AGEs) disrupt this balance mechanism. Consequently, M1 macrophage polarization persists in diabetic wounds, driving a pro-inflammatory feedback loop via NF-κB/STAT1 signaling and impeding transition to the proliferative phase (5, 8). As core effector cells of innate immunity, the switch of macrophages to the M2 phenotype has been confirmed as an effective therapeutic approach for diabetic wound healing (9, 10). Therefore, the dynamic process of imbalance and reconstruction of macrophage polarization homeostasis is significantly associated with the pathophysiology of diabetic wound healing, and regulating this polarization state is a key target for promoting diabetic wound repair currently.

Low-intensity pulsed ultrasound (LIPUS) is a non-invasive physical therapy that harnesses mechanical bioeffects from ultrasonic pulses to elicit various physiological responses. Possessing multiple therapeutic benefits, ultrasound therapy has become a research hotspot in recent years owing to its unique non-invasiveness and clinical compatibility (11). In the process of diabetic wound repair, LIPUS functions through various mechanisms: its acoustic streaming shear stress can drive the proliferation and migration of endothelial cells, thereby improving microcirculatory perfusion; meanwhile, it inhibits pro-inflammatory signaling pathways such as (nuclear factor kappa-B)NF-κB and reshapes a pro-reparative immune microenvironment (12, 13). Notably, ultrasound microbubble contrast agents (MBs) can significantly enhance the efficacy of ultrasound therapy, with the underlying mechanism that MBs strengthen the cavitation effect of LIPUS by increasing the number of cavitation nuclei and reducing the cavitation threshold (14, 15).

LIPUS can alleviate tissue inflammation by promoting the polarization of macrophages towards the M2 anti-inflammatory phenotype (11). Theoretically, ultrasound combined with microbubbles (USMB) can enhance the therapeutic efficacy of ultrasound alone. Previous studies have indicated that USMB can remodel the immune microenvironment through the recruitment of macrophage populations and the proliferation of microglia, which may be associated with the activation of CD8+ and CD4+ T cells (16, 17). Studies also suggest that ultrasound cavitation exerts anti-inflammatory effects partly through pathways like MAPK and NF-κB (13). However, the upstream signaling events that trigger the anti-inflammatory response following USMB treatment remain unknown. Using high-throughput RNA sequencing technology, we for the first time identified that the IL-17 signaling pathway plays a crucial role in USMB-mediated diabetic wound healing. Notably, this pathway serves as an upstream regulatory signal of the NF-κB inflammatory pathway, and previous studies have confirmed that targeting the IL-17 signaling pathway accelerates the process of diabetic wound healing.

Clinically, we have shown that commercially available diagnostic ultrasound combined with microbubble contrast agents can treat refractory diabetic ulcers (15). However, the mechanism of action of USMB in diabetic wound repair remains unclear. Therefore, the purpose of this study is to investigate whether USMB can induce macrophage polarization in the wounds of diabetic rats and thereby promote wound healing by alleviating inflammation. physical exams and lab results.

## 2 Materials and methods

### 2.1 Animal experiment

Male Sprague Dawley rats (4–6 weeks old, 80–120 g) were provided by Chengdu Dashuo Laboratory Animal Co., Ltd. All animals were housed in a standard laboratory animal facility with free access to food and water for one week. All animal experiments in this study were approved by the Ethics Committee of The General Hospital of Western Theater Command (No: 2023EC5-ky082).

### 2.2 Diabetic model construction

After SD rats were acclimated for one week, they were fed a high-fat and high-sugar diet for an additional 4 weeks with daily replacement of diet and water. At the end of 4 weeks, a diabetic rat model was established by a single intraperitoneal injection of streptozotocin (STZ) at a dose of 55 mg/kg. Blood glucose levels were measured using a blood glucose meter on the 3rd and 7th day of each week, and changes in body weight, food intake, and other indicators of the rats were monitored. The diabetic model was considered successful if the fasting blood glucose was > 16.8 mmol/L and the “three polys and one weight loss” symptoms persisted for more than 4 weeks. Successfully modeled rats were anesthetized with 0.3% sodium pentobarbital (40 mg/kg), and the hair on their backs was removed with depilatory cream. A full-thickness skin wound was then created using a skin punch biopsy instrument (diameter = 1.5 cm).

### 2.3 USMB treatment

According to different treatment methods, diabetic rats with successful modeling were randomly divided into 3 groups: Model group, Ultrasound (US) treatment group, and Ultrasound combined with Microbubbles (USMB) treatment group. The Model group received no intervention, the US group was treated with ultrasound alone, and the USMB group was treated with ultrasound combined with intravenous injection of microbubbles. The microbubbles (MB) were derived from the commercial ultrasound contrast agent Sonovue(Bracco International BV). According to the instructions, the contrast agent was dissolved in 5 ml of normal saline to prepare a suspension containing 59 mg of SF6 gas and 25 mg of lyophilized powder, with an MB concentration of 2×10^8^ bubbles/ml. After anesthesia with sodium pentobarbital, the rats had an acoustic coupling pad covered on the wound, and then the ultrasound probe was placed above the wound to start treatment. For USMB treatment, 1 ml of the MB suspension was injected into the rats via the tail vein. The treatment duration was 10 minutes per session, administered once every other day, with a total treatment period of 18 days. The ultrasound therapy equipment used was the SASET healthcare ultrasound diagnosis and treatment all-in-one machine, with the following parameters: frequency of 6.6 MHz, acoustic power at grade 5, pulse repetition frequency (PRF) of 100 Hz, high line density, 5 seconds of treatment, 5 seconds of interval, and pulse duration of 1200 μs.

### 2.4 Wound evaluation and tissues harvesting

The day of full-thickness skin wound modeling in diabetic rats was designated as Day 0. Wound images were captured using a digital camera on Days 0, 6, 12, and 18, respectively. Morphometric analysis of the wounds was performed using Image J software. The wound healing rate was calculated by the following formula: Wound healing rate = (Wound area on Day 0 - Wound area at the current time point)/Wound area on Day 0. Tissue samples were collected on Days 6, 12, and 18 after ultrasonic cavitation treatment, and the excised wound tissues were divided into two parts. One part was paraffin-embedded for Hematoxylin and Eosin (H&E) staining, Masson’s Trichrome (MT) staining, immunohistochemical analysis, and immunofluorescence analysis; the other part was used for quantitative real-time polymerase chain reaction (RT-qPCR), Western Blot (WB), and RNA sequencing analysis.

### 2.5 Cell culture and treatment

RAW264.7 macrophages were cultured in complete Dulbecco’s Modified Eagle Medium (DMEM) supplemented with 10% fetal bovine serum (FBS) and 1% penicillin-streptomycin, and incubated in a sterile environment (37°C, 5% CO_2_). Cell grouping and treatment methods were the same as above. Lipopolysaccharide (LPS) at 100 ng/ml was added 24 hours before cell treatment to simulate the inflammatory microenvironment of diabetic wounds.

### 2.6 H&E and MASSON staining

After collection, the tissues were embedded in paraffin and subsequently sectioned into 4 μm-thick slices for H&E staining and Masson staining. Following successful staining, high-resolution images were obtained, and the granulation tissue thickness and collagen fiber area of each rat were measured.

### 2.7 Immunohistochemistry

Paraffin-embedded wound tissues were sectioned into 4μm slices, followed by dewaxing and antigen retrieval. Subsequently, the slices were blocked with 5% bovine serum albumin (BSA) at room temperature for 1 hour. After three washes with PBS, they were incubated overnight at 4 °C with anti-CD31 antibodies (1:1000, abcam) and anti-Ki67 antibodies (1:1000, Proteintech). On the following day, the slices were removed from 4 °C and rewarmed for 1 hour. After washing, they were incubated with secondary antibody for 1 hour. Following mounting, high-resolution images were acquired using an automatic slide scanner to evaluate cell proliferation and angiogenesis.

### 2.8 Immunofluorescence

CD86 (M1 marker) and CD206 (M2 marker) were used to label M1 and M2 macrophages in wound tissues for immunofluorescence staining. After 4 μm paraffin sections were subjected to dewaxing, antigen retrieval, and BSA blocking, they were incubated overnight at 4 °C with primary antibodies against CD206 (1:200, Proteintech) and CD86 (1:50, Proteintech). On the following day, following rewarming, the sections were labeled with secondary antibodies for 1 hour to track macrophages. DAPI stained the cell nuclei blue, CD206 was labeled green with an IgG Alexa Fluor 488-conjugated secondary antibody, and CD86 was labeled red with an IgG Alexa Fluor 647-conjugated secondary antibody. Finally, stained images were acquired under a fluorescence microscope (Leica).

### 2.9 RT -qPCR

Total RNA was extracted from wound tissue and the RAW264.7 cell line using TRIzol, followed by assessment of RNA integrity and quality. cDNA was synthesized using a reverse transcription kit (Sangon Biotech, China). β-actin was used as the reference gene, and the primer sequences for each target gene are listed in **Table 1**. RT-qPCR was performed using SGExcel FastSYBR Mixture (Sangon Biotech) on a BioRadCFX Manager detection system according to the predetermined protocol. The 2^−ΔΔCt method was applied to calculate the relative expression levels of target genes in the samples.

**Table 1:** Primer names and sequences.

Primer	Forward primer	Reverse primer
B-actin	CCACTGCCGCATCCTCTT	GTCAGCAATGCCTGGGTA
TNF-α	GGTGATCGGTCCCAACAAGGA	CACGCTGGCTCAGCCACTC
IL-17B	CCAGCTGAGGAACAGCTCTGA	AGACAGGCTTCTCTTGTTGGATAAC
Arg1	CTCCAAGCCAAAGTCCTTAGAG	AGGAGCTGTCATTAGGGACATC
Cxcl1	ACCCAAACCGAAGTCATAGCC	TGGGGACACCCTTTAGCATCTT
Cxcl2	CAATGCCTGACGACCCTACC	TCAGTTAGCCTTGCCTTTGTTC
Cxcl3	GCCCCAAGGTGGAAGTCATA	TTTGTTTTCTTATTTTCACTGCCCA
MAPK13	GGCGGCCAAATCCTACAT	GGGAAAAGCTGTGTGAAATCCT
MMP8	CCATGGATCCAGGTTACCCCACT	TGTGGTCCACTGAAGAAGAGGAAGA
MMP9	GGATGTTTTTGATGCCATTGCTG	CCACGTGCGGGCAATAAGAAAG
IL-1β	ACCCAAGCACCTTCTTTTCCTT	TGCAGCTGTCTAATGGGAACAT
IL-10	GGTTGCCAAGCCTTGTCAGAA	GCTCCACTGCCTTGCTTTTATT
IL-6	GACAGTGCATCATCGCTGTTCATA	AGTCGGAGGCTTAATTACATATGTTC
iNOS	GGTATGCTGTGTTTGGCCTT	GCAGCCTCTTGTCTTTGACC

### 2.10 Western blotting

Total protein was extracted from wound tissue using a total protein extraction kit (Beyotime, China), and the protein concentration was determined by the BCA method (solarbio, China). A total of 50 μg protein per sample was separated by 10% sodium dodecyl sulfate–polyacrylamide gel electrophoresis (SDS-PAGE) and transferred onto a polyvinylidene fluoride (PVDF) membrane (Millipore, USA). The membrane was blocked with 5% skim milk prepared in Tris-buffered saline with Tween (TBST), followed by overnight incubation with primary antibodies against IL-17B (1:800, BOSTER), NF-κB (1:2000, Proteintech), TNF-α (1:800, BOSTER), and IL-1β (1:1000, Proteintech). The next day, the membrane was incubated with horseradish peroxidase (HRP)-conjugated anti-rabbit secondary antibody. Finally, protein bands were visualized using an ECL reagent (solarbio, China) and exposed in a darkroom with a chemiluminescence imaging system.

### 2.11 RNA sequencing and bioinformatics analysis

According to standard protocols, total RNA was isolated from diabetic wound tissues on day 6 after USMB treatment using TRIzol Reagent (Invitrogen, USA), followed by rigorous quality control of the RNA samples. The integrity of RNA was accurately assessed using the Agilent 2100 bioanalyzer. mRNA was enriched from total RNA using Oligo dT magnetic beads, fragmented, and then first-strand cDNA was synthesized with random hexamer primers, followed by second-strand synthesis. After steps including end repair, adenylation, adapter ligation, fragment selection, amplification, and purification, library construction was completed. Qualified libraries were quantified and pooled based on effective concentration and required data volume, followed by sequencing on the Illumina platform. Sequencing images were base-called and converted into FASTQ format sequence data (reads), containing sequence information and quality scores. Raw data containing adapter contamination, low-quality reads, and unrecognized bases (N) may compromise subsequent analyses. To ensure the quality and reliability of data analysis, raw data were filtered using Fastp (version 0.23.1) for quality control.

### 2.12 Statistical analysis

Experimental data were statistically analyzed using GraphPad Prism 10 software, and results are presented as mean ± SD following confirmation of the assumptions of normal distribution and homogeneity of variance. Comparisons among multiple groups were performed using one-way analysis of variance (one-way ANOVA); if a significant overall difference was detected, pairwise comparisons between groups were further conducted via the Tukey *post-hoc* test. At least three independent experiments were carried out, and a p-value<0.05 was considered statistically significant.

## 3 Results

### 3.1 Induction of the diabetic rat model

**Figure 1:**
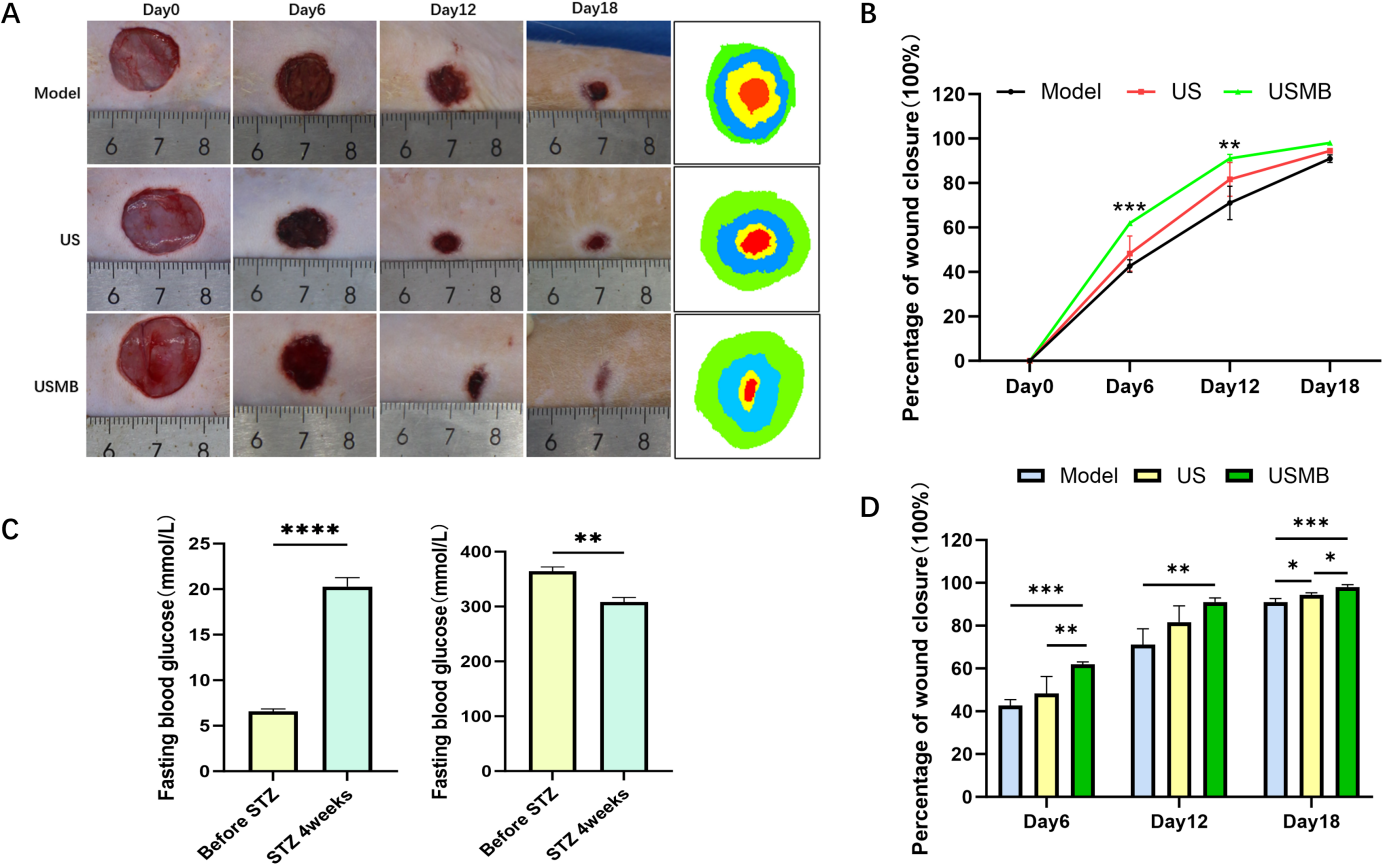
Establishment of diabetic rat models and changes in wound healing following USMB treatment. **(A)** Wound healing images and superimposed wound maps of the three groups on days 0, 6, 12, and 18; **(B, D)** Line graph and bar chart showing wound healing areas of the three groups on days 0, 6, 12, and 18; **(C)** Changes in body weight and blood glucose levels before and 4 weeks after STZ injection. Data are presented as mean ± SD, n=4. *p < 0.05, **p< 0.01, ***p< 0.001, ****p < 0.0001.

In this study, a diabetic rat model was induced using a high-fat diet combined with streptozotocin (STZ) administration. Following successful induction of diabetes, a full-thickness wound healing model was created on the dorsal skin. Parameters were compared between pre-injection baseline and 4 weeks post-STZ injection. Four weeks post-injection, the rats exhibited classic diabetic symptoms: polydipsia, polyphagia, and polyuria. Moreover, a declining trend in body weight was observed 4 weeks after STZ injection, consistent with the weight loss commonly seen in patients with type 2 diabetes. Fasting blood glucose levels measured after a 12-hour fast revealed a significant increase in blood glucose in rats 4 weeks after STZ injection, with all values exceeding 16.8 mmol/L (**Figure 1C**), demonstrating the successful induction of the diabetic rat model through the combination of a high-fat diet and intraperitoneal STZ injection.

### 3.2 USMB promotes wound healing in diabetic rats

Wound areas were photographed and measured on days 0, 6, 12, and 18, and the wound healing rate was calculated using the aforementioned formula to evaluate the effect of USMB on wound healing in diabetic rats (**Figure 1A**). Compared to the Model group, both the US and USMB groups showed significantly higher wound healing rates, with the most pronounced effect observed on day 6 (**Figures 1B, D**). On day 6, the wound healing rates of the Model, US, and USMB groups were 42.6%, 48.3%, and 62.0%, respectively. By day 12, the healing rate in the USMB group reached 91.1%, which, although the rate of increase had slowed, remained significantly higher than that in the Model (71.1%) and US (81.6%) groups. On day 18, the wound healing rates were 98.1% in the USMB group, 94.4% in the US group, and 90.9% in the Model group. These results demonstrate that USMB treatment promotes wound healing in diabetic rats.

### 3.3 USMB improves wound granulation tissue proliferation and collagen deposition

**Figure 2:**
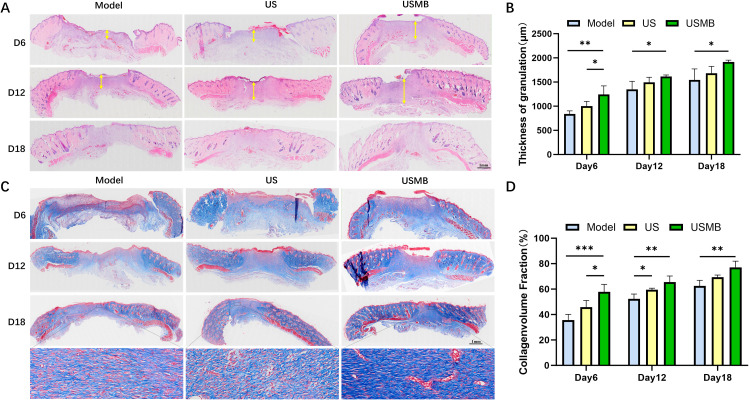
Histopathological staining of wound tissues in diabetic rats. **(A)** H&E staining of wound tissues from the USMB group, US group, and Model group; yellow arrows indicate the thickness of granulation tissue. **(B)** Granulation tissue thickness of diabetic wounds in the three groups on days 6, 12, and 18. **(C)** Masson staining of wound tissues from each group. **(D)** Statistical graph of collagen fiber area in each group at three time points: days 6, 12, and 18. Data are presented as Mean ± SD, n = 4; *p< 0.05, **p< 0.01, ***p< 0.001.

H&E staining revealed that re-epithelialization was observed in the USMB group as early as day 6. The extent of re-epithelialization in the US group was less than that in the USMB group, while it was least apparent in the Model group (**Figure 2A**). By days 12 and 18, all three groups exhibited notable re-epithelialization, with the USMB group still showing the most significant improvement and being closest to normal skin. Statistical analysis of granulation tissue thickness indicated that on day 6, the USMB group was significantly higher than the other two groups; although the increase in the USMB group slowed on days 12 and 18, it remained the highest among the three groups (**Figure 2B**). This suggests that USMB treatment can enhance granulation tissue thickness in diabetic wounds, promote re-epithelialization, and accelerate healing. As shown in **Figures 2C, D**, on day 6, the USMB group exhibited the largest collagen fiber area. Both the US and USMB groups showed collagen deposition, whereas it remained relatively scant in the Model group. By days 12 and 18, all three groups displayed varying degrees of collagen deposition, yet the collagen fiber area in the USMB group consistently surpassed that of the other two groups. Moreover, compared to the Model and US groups, the USMB group demonstrated denser collagen fiber arrangement, greater quantity, and a closer resemblance to normal tissue. These results indicate that USMB treatment facilitates collagen deposition in diabetic rat wounds and promotes the healing of diabetic wounds.

### 3.4 USMB promotes angiogenesis and cell proliferation in wound tissues

**Figure 3:**
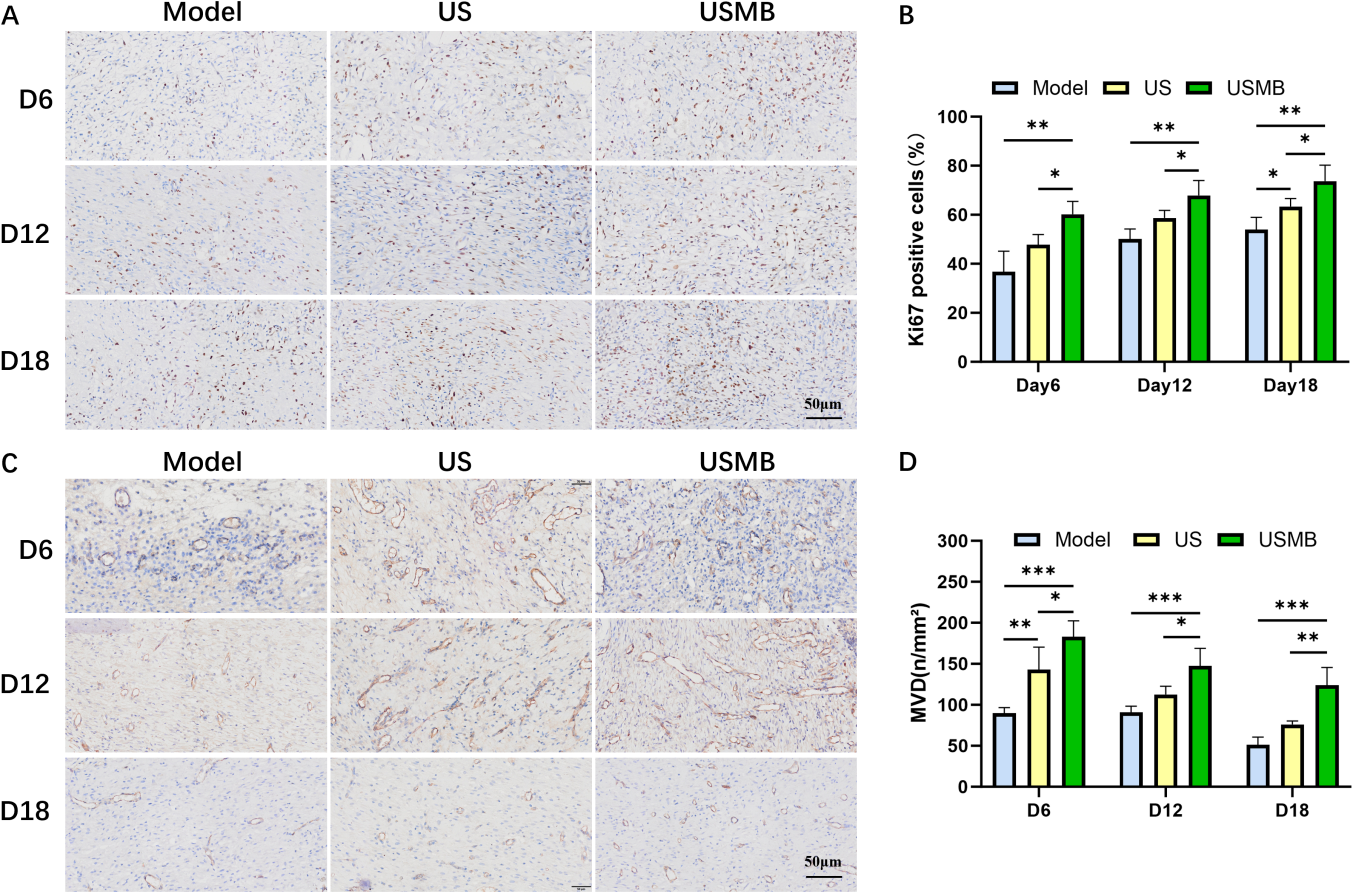
Immunohistochemical staining of wounds in diabetic rats. **(A)** Representative images of Ki67 immunohistochemical staining in healing tissues from three experimental groups; **(B)** Quantitative analysis of Ki67-positive cells across groups; **(C)** Immunohistochemical staining of CD31 in diabetic wounds; **(D)** Statistical results of CD31-positive expression. Data are presented as mean ± SD (n = 4); *p < 0.05, **p < 0.01, ***p < 0.001.

CD31 serves as a marker for neovascularization. Immunohistochemical staining for CD31 in diabetic wounds is shown in **Figure 3A**, and the corresponding quantitative results are presented in **Figure 3B**. On day 6, CD31-labeled neovessels reached their peak level, with both the USMB group and the US group exhibiting significantly higher CD31 expression than the Model group. By day 12, the number of new blood vessels began to decline; however, CD31 expression in the USMB group remained higher than that in the other two groups. This trend continued until day 18, when CD31 levels in all three groups dropped to relatively low levels, though the USMB group still showed the highest CD31 content. These results suggest that USMB treatment accelerates wound closure, at least in part, by enhancing angiogenesis. Ki67 staining results are displayed in **Figures 3C, D**. On day 6, the percentage of Ki67-positive cells in the USMB group was significantly higher than that in the other two groups, reaching 60.2%. By day 12, the proportions of Ki67-positive cells in the Model and US groups increased to 50.1% and 58.7%, respectively, while the USMB group maintained a higher level at 67.9%. On day 18, the USMB group continued to exhibit the highest percentage of Ki67-positive cells (73.7%), surpassing the Model and US groups (53.9% and 63.4%, respectively). Overall, Ki67 expression was most pronounced in the USMB group, suggesting that USMB enhances cell proliferation during wound healing in diabetic rats.

### 3.5 USMB treatment attenuates inflammation and regulates macrophage polarization

**Figure 4:**
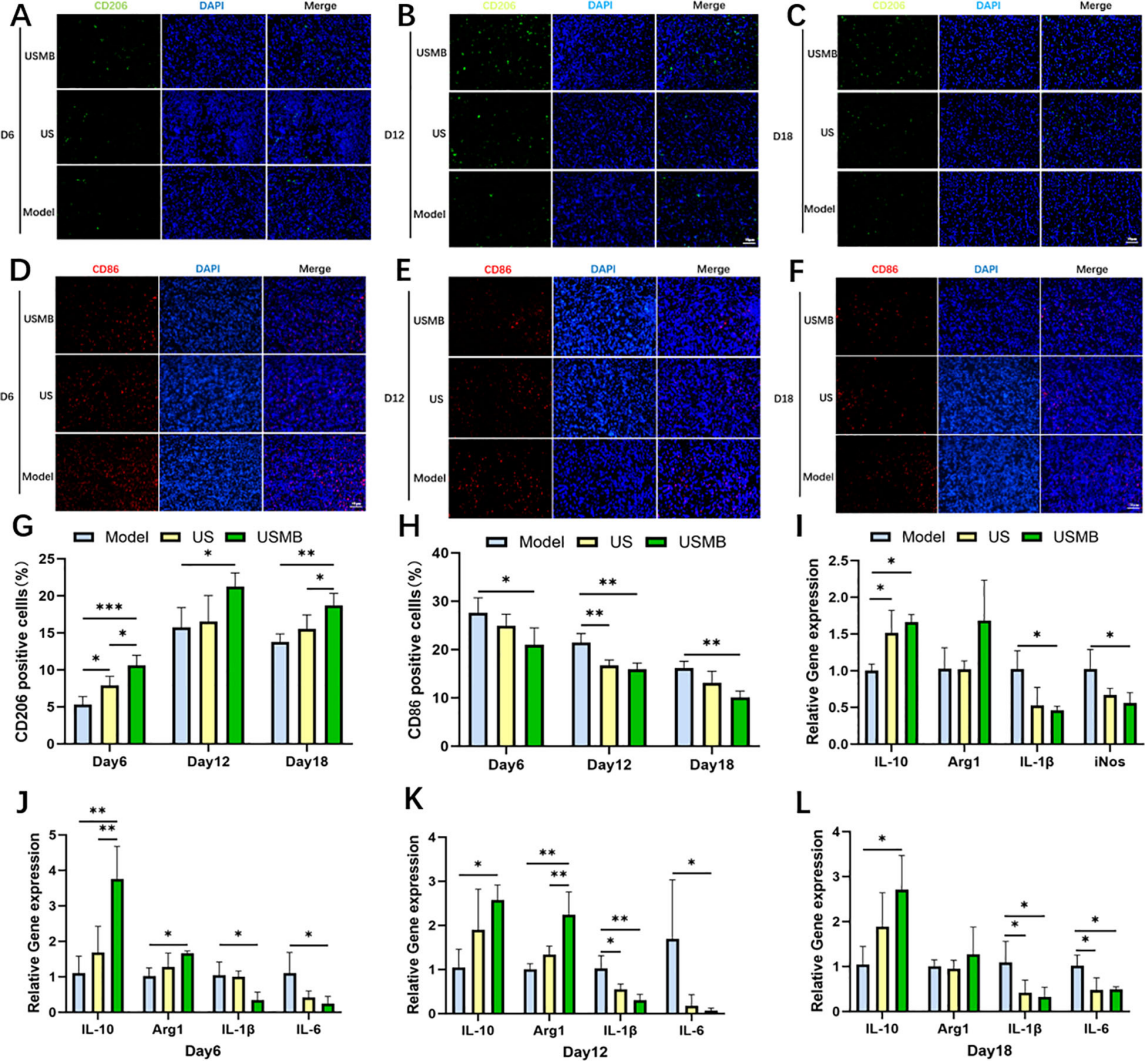
Immunofluorescence staining of diabetic wound healing tissues and RT-qPCR analysis of mRNA relative expression of macrophage-related inflammatory factors. **(A-F)** Immunofluorescence staining images of healed wound tissues in diabetic rats: CD206 is shown in green, CD86 in red, and nuclei in blue; **(G, H)** Statistical graphs of immunofluorescence staining results from the three groups; **(I)** Statistical graph of inflammatory factor mRNA expression in RAW264.7 macrophages after 24-hour intervention; **(J-L)** Statistical graphs of inflammatory factor mRNA expression in wound tissues of the three groups of diabetic rats at three time points. Scale bar = 50μm; Data presented as Mean ± SD, n = 4; *p < 0.05, **p< 0.01, ***p < 0.001.

To examine the impact of USMB on macrophage dynamics, immunofluorescence staining was performed immunofluorescence staining for CD206 (**Figures 4A–C**) and CD86 (**Figures 4D–F**) in the wound tissue. As shown in **Figure 4G**, CD206 expression exhibited an initial increase followed by a decrease across all three groups, peaking on day 12. On day 6, the overall level of CD206 was relatively low, although it was higher in the USMB group compared to the other two groups, and showed the greatest increase by day 12. By day 18, CD206 levels in all groups began to decline, suggesting that wound repair was nearing completion. From **Figure 4H**, it can be observed that CD86 expression in all three groups peaked on day 6 and subsequently decreased. On day 12, CD86 in the USMB group was significantly lower than in the Model group and was comparable to the US group, though both were lower than the Model group. By day 18, CD86 in the USMB group dropped to the lowest level, indicating that the wound healing status was approaching that of normal diabetic skin tissue.

To further examine the expression of macrophage-related inflammatory cytokines, we performed PCR assays for M1 macrophage markers (IL-6, IL-1β, iNOS) and M2 macrophage markers (IL-10, Arg1). As shown in **Figures 4J–L**, starting from day 6, the pro-inflammatory cytokines IL-6 and IL-1β in the USMB group were significantly reduced compared to the US and Model groups, while the anti-inflammatory cytokines IL-10 and Arg1 were markedly increased, with IL-10 showing the most pronounced elevation. This indicates that USMB treatment effectively suppressed the inflammatory response and promoted the transition from the inflammatory phase to the proliferative phase in wound healing. By day 12, the expression of anti-inflammatory cytokines IL-10 and Arg1 in the USMB group reached relatively high levels, and the pro-inflammatory cytokine IL-6 decreased most significantly, suggesting that inflammation in the diabetic rat wounds was effectively controlled. On day 18, compared to the US and Model groups, the USMB group exhibited a decline in anti-inflammatory cytokine expression and a slight increase in pro-inflammatory cytokine levels, indicating that the wounds were approaching the final stages of repair following USMB treatment. To preliminarily verify the direct effect of USMB on the inflammatory response of macrophages at the cellular level, we treated LPS-stimulated RAW264.7 cells with USMB for 24 hours and detected the mRNA expression of related factors. As shown in **Figure 4I**, USMB treatment effectively inhibited the mRNA expression of the pro-inflammatory factors IL-6 and IL-1β, while upregulating that of the anti-inflammatory factors IL-10 and Arg1. These results preliminarily suggest that USMB can regulate the inflammatory response-related gene expression program of macrophages(**Figure 4I**). These findings suggest that USMB therapy may be associated with promoting the polarization of macrophages from the M1 to the M2 phenotype, which is consistent with the observed inhibition of wound inflammatory responses and accelerated wound healing.

### 3.6 RNA-seq analysis reveals the mechanism by which USMB modulates macrophage polarization in diabetic wounds

**Figure 5:**
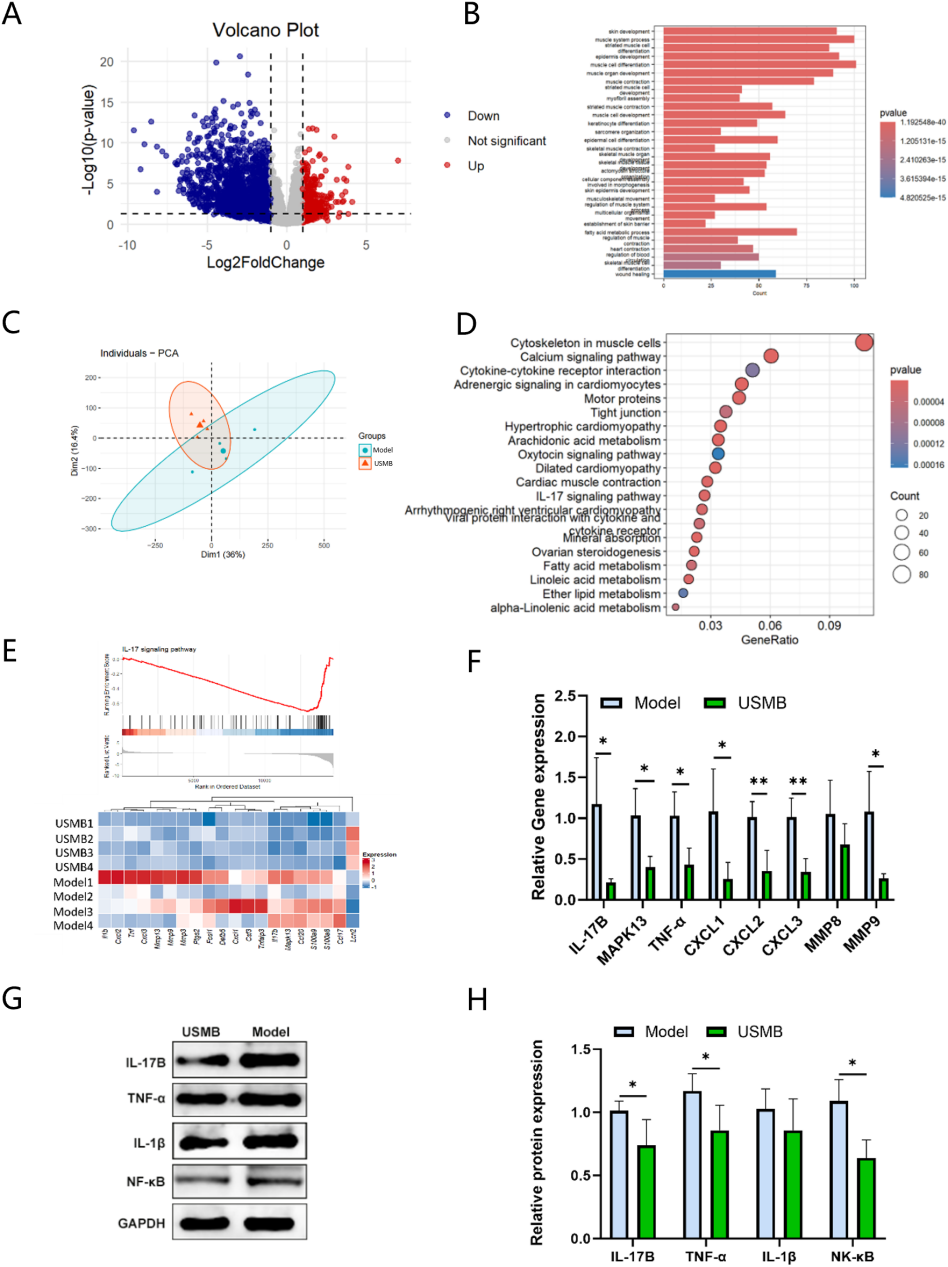
Transcriptome sequencing results and validation. **(A)** Volcano plot of differentially expressed genes between the USMB group and the Model group; **(B)** GO enrichment analysis; **(C)** Principal component analysis (PCA) plot; **(D)** KEGG enrichment analysis of differentially expressed genes; **(E)** Gene set enrichment analysis (GSEA) and corresponding heatmap; **(F)** PCR validation of selected differentially expressed genes associated with the IL-17 signaling pathway based on sequencing results; **(G, H)** Validation of IL-17 signaling pathway: protein expression levels of IL-17B, TNF-α, IL-17β, and NF-κB, along with quantitative statistical results. Data are presented as Mean ± SD, n= 4; *p< 0.05, **p< 0.01.

To preliminarily explore the molecular mechanisms by which USMB therapy promotes macrophage polarization in diabetic wounds, we performed gene expression profiling via RNA-seq to identify potential regulatory pathways. PCA results demonstrated a significant intergroup sample divergence. (**Figure 5C**). **Figure 5A** shows the volcano plot of differentially expressed genes. A total of 1,725 differentially expressed genes were identified in wound tissues between the USMB and Model groups, including 296 upregulated and 1,429 downregulated genes. GO/KEGG enrichment analysis revealed that USMB treatment primarily downregulated genes associated with signaling pathways mediated by inflammation, chemokines, lipid metabolism, etc. (**Figures 5B, D**). GSEA enrichment analysis indicated significant enrichment of the IL-17 signaling pathway with a downregulated trend (**Figure 5E**). Combined with KEGG enrichment analysis, it was found that the IL-17 signaling pathway may play an important role in USMB treatment promoting diabetic wound healing. The lower part of **Figure 5E** shows a heatmap of genes closely related to this pathway identified based on IL-17 enrichment analysis. These genes represent differentially expressed genes in the IL-17 signaling pathway between the USMB and Model groups, including IL-1β, CXCL2, TNF-α, CXCL3, MMP13, MMP9, MMP3, Ptgs2, FOSL1, Defb5, CXCL1, Cst3, Tnfaip3, IL-17B, MAPK13, Ccl20, S100a9, S100a8, Ccl17, and Lcn2. To validate the effect of USMB treatment on the IL-17 signaling pathway, we performed RT-qPCR to detect the expression of IL-17 and its downstream related genes in wound tissues on day 6, including: IL-17B, MMP9, MMP8, Mapk13, TNF-α, Cxcl1, Cxcl2, Cxcl3, and IL-1β. As shown in **Figure 5F**, the expression of the aforementioned genes was significantly decreased after USMB treatment, which is consistent with the sequencing results. Furthermore, to further clarify how USMB treatment mediates macrophage polarization through the IL-17 signaling pathway, we performed Western blot analysis on IL-17 and genes along its pathway axis. The results showed that the protein expression levels of IL-17B, NF-κB, IL-1β, and TNF-α in the USMB group were significantly lower than those in the Model group (**Figures 5G, H**), suggesting that the process by which USMB treatment regulates the IL-17 signaling pathway to promote macrophage polarization may be achieved by suppressing the IL-17B/NF-κB/TNF-α, IL-1β pathway axis.

## 4 Discussion

Ultrasound imaging is one of the most widely used diagnostic modalities globally, and its therapeutic applications have also progressed substantially in recent years. LIPUS was approved by the FDA as early as 2017 for accelerating fracture healing and treating specific tendon and cartilage disorders (18, 19). Substantial evidence suggests that ultrasound therapy can inhibit inflammation, promote angiogenesis, and facilitate tissue repair and regeneration (11, 20). This study addresses the clinical challenge of diabetic wound healing and adopts a strategy of exploratory transcriptomics combined with multi-group and multi-time-point validation. Building on the confirmed therapeutic efficacy of USMB, we screened for potential targets and validated their functions experimentally, thereby elucidating the pro-healing mechanisms of USMB.

The pro-healing effects of USMB were initially evident in the accelerated overall wound repair process. Compared with the model group and the ultrasound-only group, the USMB group exhibited significantly higher wound healing rates on days 6, 12, and 18, with a distinct advantage observed especially in the early stage of treatment (day 6). Histopathological analysis confirmed that the thickness of granulation tissue in the USMB group was greater than that in the US group and Model group on day 6. Notably, granulation tissue in the Model group had not yet fully formed at this time, indicating that USMB treatment laid a more favorable foundation for subsequent tissue repair and reconstruction of the wound. Studies have shown that mechanical forces generated by ultrasound cavitation can promote the proliferation and migration of fibroblasts (21). When microbubbles injected via the tail vein circulate in the bloodstream, they can resonate with ultrasonic energy, act on cell pores, and induce the local release of growth factors such as VEGF and FGF, thereby accelerating granulation formation (15). The deposition of collagen fibers is also a crucial process in wound healing; an increase in its content can enhance the strength and stability of wound tissue, facilitating wound healing. Masson’s trichrome staining indicated that USMB treatment promoted collagen fiber expression in wounds, likely due to accelerated collagen deposition. Research by other scholars has indicated that the transmission of mechanical forces generated by ultrasonic energy can activate the TGF-β1 pathway in fibroblasts, thereby promoting collagen synthesis (22). Additionally, a study by Hu et al. found that low-intensity ultrasound stimulation can improve the structure of collagen fibers (23), which is consistent with our finding that collagen arrangement in wounds becomes tighter after USMB treatment.

Cell proliferation is fundamental to injury repair, a process coordinated by multiple cell types and involving migration, proliferation, matrix deposition, inflammation, and angiogenesis. Studies have demonstrated that the ultrasonic cavitation effect can activate certain intracellular signaling pathways and enhance cellular proliferative capacity (24). Results from Ki67 immunohistochemical staining revealed that the number of Ki67-positive cells in the USMB group was highly expressed at all time points, confirming that USMB treatment significantly promotes cell proliferation during wound healing in diabetic rats. The proliferative phase of wound healing involves the participation of various cell types, including endothelial cells, fibroblasts, and myofibroblasts, which collectively act to facilitate wound repair (25). During the repair process, endothelial cells form new blood vessels, supplying oxygen and nutrients essential for wound healing. A study by Chen et al. showed that LIPUS can stimulate wounds to release vascular endothelial growth factors (VEGF), promoting the proliferation and migration of endothelial cells and thereby facilitating angiogenesis (12). CD31 is a marker of neovascularization. Our study found that CD31 expression in the USMB group was higher than that in the US group and Model group on days 6, 12, and 18, indicating that USMB promotes neovascularization. Moreover, CD31 expression peaked on day 6 and gradually declined thereafter, consistent with the progression from proliferative to remodeling phases in wound healing.

Inflammation regulation represents another critical mechanism underlying USMB-mediated wound healing. Persistent inflammation in diabetic wounds is primarily attributed to the excessive infiltration of M1 macrophages and the massive release of pro-inflammatory cytokines (26). The core regulatory mechanism of wound repair lies in establishing a dynamic balance between inflammation resolution and tissue regeneration. Research findings have demonstrated the dual effects of USMB therapy in diabetic wounds: while inhibiting pro-inflammatory cytokines, USMB activates M2 macrophage function by upregulating repair-related genes (Arg1, IL-10).

In the present study, we found that USMB significantly decreased the expression of the M1 macrophage marker CD86, while upregulating the level of the M2 marker CD206, accompanied by the downregulation of pro-inflammatory factors IL-6 and IL-1β, as well as the upregulation of anti-inflammatory factors IL-10 and Arg1. Further time-course analysis revealed that USMB exhibited dynamic characteristics: the inhibitory effect on the pro-inflammatory factor IL-6 was most pronounced on day 6, and the expression of the repair factor Arg1 was significantly increased on day 12. These findings suggest that USMB can dynamically regulate macrophage function and facilitate the transition from the inflammatory phase to the proliferative phase. Notably, Arg1 supports collagen synthesis and angiogenesis by promoting arginine metabolism (27),which is consistent with our previously observed results of collagen deposition and neovascularization, thereby validating the role of USMB in coordinating the balance between inflammation resolution and tissue regeneration.

Excessive activation of the IL-17 signaling pathway is a crucial driver of inflammation in diabetic chronic wounds (28). This study is the first to reveal via RNA-seq analysis that USMB treatment inhibits the IL-17 signaling pathway, in which the expression of multiple key genes including CXCL2, TNF-α, CXCL3, MMP8, MMP9, CXCL1, IL-17B and MAPK13 is significantly downregulated. Among these, the expression change of IL-17B is particularly prominent and exhibits a trend of synergistic downregulation with core inflammatory factors such as TNF-α and IL-1β, suggesting that it may play a pivotal role in the inflammatory regulation of diabetic wounds. Subsequent validation using qPCR and Western Blot confirmed that USMB can significantly reduce the expression of IL-17B and its downstream molecules NF-κB and TNF-α, thereby suggesting that USMB may regulate macrophage polarization and inflammatory responses by targeting the IL-17B/NF-κB/TNF-α axis. Through the synergistic regulation of these genes, we can achieve a more comprehensive understanding of the USMB-mediated anti-inflammatory and reparative balance mechanism. For instance, the inhibition of MMP9 and CXCL2 suggests that USMB can improve the repair microenvironment by reducing collagen degradation and neutrophil infiltration (29, 30). Additionally, the downregulation of MAPK13 may inhibit p38 MAPK phosphorylation in macrophages, thereby blocking signals associated with M1 macrophage polarization (31). These findings are consistent with the research conclusions of Zheng et al., who demonstrated the involvement of the MAPK13/p38 signaling pathway in macrophage polarization (32). Moreover, this study expands the mechanistic understanding of this signaling module in ultrasound therapy from the perspective of USMB-mediated regulation of the IL-17 pathway. At the protein level, Western blot (WB) results showed that the expression levels of IL-17B, NF-κB, TNF-α, and IL-1β in wound tissues decreased after USMB treatment. This indicates that USMB treatment may inhibit the activation of the IL-17B/NF-κB/TNF-α inflammatory signaling axis, block the polarization program of M1 macrophages, and thereby drive the transformation of macrophages toward the pro-reparative M2 phenotype. Studies by Kusuyama et al. and Yi et al. have separately reported the regulatory effects of low-intensity pulsed ultrasound (LIPUS) on inflammatory factors (such as IL-1β and TNF-α) and the NF-κB pathway (33, 34), which are partially consistent with the results of this study. Furthermore, the present work further reveals that USMB may exert mechanical stimulation generated by microbubble cavitation at the level of IL-17 receptors, thereby regulating NF-κB signal transduction. This complements previous studies on the anti-inflammatory mechanisms of ultrasound cavitation therapy and provides a novel perspective on the immunomodulatory effects of USMB in the treatment of diabetic wounds.

The findings of this study provide experimental evidence for ultrasound combined with microbubbles (USMB) in the treatment of diabetic wounds and indicate its potential for clinical translation. The ultrasound parameters employed in this study are derived from FDA-approved commercial diagnostic devices, with their low-intensity pulsed output being suitable for human tissue; additionally, Sonovue, the microbubble contrast agent, is itself a well-established clinical formulation, which provides the safety and technical underpinnings for this therapeutic regimen (18, 35). In terms of therapeutic efficacy, USMB exhibited superior capacity for promoting wound healing, enhancing angiogenesis and modulating the immune microenvironment compared with ultrasound monotherapy, suggesting that the addition of microbubbles may exert a synergistic potentiation effect. Although microbubble formulations have a favorable short-term safety profile, future research is required to systematically investigate the risks associated with their long-term and repeated administration in clinical practice, refine and optimize the therapeutic parameters based on the physiological characteristics of human wounds, and ultimately establish the clinical position and therapeutic value of USMB in the treatment of diabetic non-healing wounds through prospective clinical trials.

## 5 Conclusions

This study is the first to combine RNA-seq with functional validation to elucidate the mechanism by which USMB may regulate macrophage polarization and promote diabetic wound healing by inhibiting the IL-17 signaling pathway, and to identify the IL-17B/NF-κB/TNF-α axis as its potential functional target. These discoveries not only provide novel therapeutic approaches for the treatment of diabetic ulcers but also establish a theoretical framework for mechanistic investigations into the biological effects of ultrasound.

### 5.5 Limitations and future directions

Limitations and Future Directions of the Study: ① The transcriptome sequencing in this study was performed for preliminary exploratory purposes. Although this technique confers the advantage of high throughput, its sample size (n=4) limits the statistical power of differential gene analysis, and an expanded cohort will be needed to validate the key targets in future research. ② In this study, the conclusion regarding macrophage polarization is primarily based on detections at the bulk tissue level. Future studies need to validate the specific regulatory effect of USMB on macrophages using flow cytometry, immune cell sorting, or single-cell RNA sequencing (scRNA-seq) techniques. ③In future investigations, we can construct IL-17B knockout or inhibitor-treated models to validate the core regulatory effects of this signaling axis on USMB-mediated macrophage polarization and wound healing, while clarifying the specific functional targets of IL-17B or its downstream adaptor proteins, thereby refining the mechanistic network.

## Data Availability

All data generated and analyzed during this study are publicly available in designated stable repositories. The transcriptome sequencing data of this study are deposited in the NCBI BioProject database under the accession number PRJNA1398177, with the direct access link: https://www.ncbi.nlm.nih.gov/bioproject/PRJNA1398177. The experimental data of this study are available at the direct access link: https://www.jianguoyun.com/c/sd/1c1bdf1/15b0167499b11742.
